# Self-Perceived Prevalence of Functional Ankle Instability and Associated Factors Among Male Volleyball Players in Qassim Region

**DOI:** 10.3390/medicina62020387

**Published:** 2026-02-16

**Authors:** Maram Ibrahim Mebrek AlMebrek, Salma Abdulmohsen Altoyan, Ahmad Alanazi, Msaad Alzhrani, Sultan A. Alanazi, Mahamed Ateef

**Affiliations:** 1 Department of Physical Therapy, Buraydah Central Hospital, Buraydah 52361, Saudi Arabia; mialmebrek@moh.gov.sa; 2 Department of Physical Therapy, King Fahad Specialist Hospital, Buraydah 52366, Saudi Arabia; saaltoyan@moh.gov.sa; 3 Department of Physical Therapy & Health Rehabilitation, College of Applied Medical Sciences, Majmaah University, Al Majmaah 11952, Saudi Arabia; aalanazi@mu.edu.sa (A.A.); m.alzhrani@mu.edu.sa (M.A.); sa.alanazi@mu.edu.sa (S.A.A.)

**Keywords:** functional ankle instability, Ar-IdFAI, scale, volleyball players, Saudi Arabia

## Abstract

*Background and Objectives*: Functional Ankle Instability (FAI) is a sequela of ankle sprains; however, its associated variables in volleyballers have not been studied. This study aimed to determine the prevalence of FAI and the association between FAI and its associated variables in volleyball players. *Materials and Methods*: An observational study with a sample size of 128 male volleyballers, aged 18 years and older, was conducted using the Arabic-Identification of Functional Ankle Instability (Ar-IdFAI) questionnaire. The prevalence of FAI was analyzed in terms of frequency and percentage. The Mann–Whitney U test, Spearman’s test, and *t*-test were used to analyze the associations between the demographic variables and the categorical variables, and a logistic regression model was applied to identify the independent associations with FAI. Statistical significance was set at *p* < 0.05. *Results*: The prevalence of FAI in the sample was 44.53%. Bivariate analysis and the regression model indicated no significant direct association between FAI and age, Body Mass Index (BMI), playing duration, weekly training hours, or limb dominance in this sample. Conversely, historical injury burden showed strong and statistically significant associations with FAI (Cramér’s V = 0.59–1.00), with “giving way” demonstrating perfect separation. The logistic regression model showed an acceptable fit (*p* = 0.676) and moderate explanatory power (Nagelkerke R^2^ = 0.540), with excellent discriminatory performance Area Under the Curve (AUC = 0.855) driven primarily by injury-related variables. *Conclusions*: FAI is highly prevalent among male volleyball players and is linked to injury history rather than demographic or training characteristics. Injury-related characteristics, including previous ankle injury, reinjury, and episodes of ankle “giving way”, demonstrated strong associations with the presence of Functional Ankle Instability, to be interpreted as descriptive associations rather than a causal link due to methodological structure.

## 1. Introduction

Volleyball is one of the top five most popular sports worldwide. Although it seems like an upper limb dominant sport players commonly develop musculoskeletal injuries [1,2], with high prevalence of knee and ankle injuries being reported (Canada (20.9%, 14%), Finland (24.3%, 12.5%), Hong Kong (27%, 11.2%), and Saudi Arabia (27%, 12%) [3,4]); out of these, the ankle joints are the most injured, as the players frequently jump and land, stressing the ankle joints [5,6]. Factors such as intense physical training, high frequency, and long experience in volleyball players may often contribute to sports-specific injuries.

A review highlighted that ankle injuries were common among volleyball players and recommended that preventive measures be applied to reduce such injuries [6,7]. A previous study from Saudi Arabia also reported that ankle injuries among male high school students in Riyadh were in the range of 14–34.7% [8], warranting a deeper probe into the phenomenon of ankle injuries in volleyball players.

Chronic ankle instability (CAI) is a broad clinical condition encompassing recurrent ankle sprains, episodes of perceived instability, and residual functional deficits following an initial ankle injury. Within this spectrum, Functional Ankle Instability (FAI) refers specifically to subjective symptoms of ankle instability, such as recurrent “giving way,” pain, or insecurity during activity, in the absence of overt mechanical laxity. FAI is generally associated with proprioceptive deficits and leads to episodes of joint instability, which can only be subjectively evaluated at one point in time [9,10]. Those with FAI often feel that their ankles give way during regular sports [11]; this type of instability affects approximately 40–72% of athletes who have experienced earlier ankle sprains [12,13,14]. FAI can also reduce motor function while playing sports and make individuals prone to new injuries of the lateral ligament of the ankle [15].

Several studies have indicated that FAI might relate to self-reported proprioceptive deficiencies and previous ankle injuries [16,17,18,19,20,21], as it is commonly characterized by a history of recurrent ankle sprains leading to difficulties in performing functional or athletic activities [22]. Therefore, comprehensive assessment, examination, and evaluation are necessary for successful prevention and management [23].

Several patient-reported outcome measures (PROMs) have emerged as common instruments for assessing ankle instability [24,25]. Particularly, the Identification of Functional Ankle Instability (IdFAI) questionnaire [26], out of all, focuses on distinguishing between stable and unstable ankles and can also be used for evaluative purposes, based on subjective reporting and recall. The psychometric properties of the scale are also within acceptable limits, making it a simplified subjective FAI evaluation tool with just ten questions grouped under initial injury details and instability history [26]. An Arabic version of the IdFAI questionnaire was designed to identify the criteria necessary for inclusion in the FAI cohort [27].

It is vital to understand FAI and the associated variables that make volleyball players more susceptible to ankle instability. Although injury rates among athletes have been documented in the literature, nothing is known about the prevalence and contributing factors of FAI in Saudi Arabian volleyball players, including national league and top players. This study is primed at investigating the prevalence of FAI and determining the association between injury-associated variables, among volleyball players in the Qassim region of Saudi Arabia. By descriptively analyzing the burden of FAI and its associated characteristics in volleyball players, this study aims to contribute to the existing literature and support the development of targeted assessment, preventive, and rehabilitative strategies to reduce the progression of ankle instability and enhance athletic performance.

## 2. Materials and Methods

### 2.1. Study Design, Setting, and Ethical Approval

This cross-sectional observational study was conducted from May to July 2024 in eight volleyball clubs in the Qassim region of Saudi Arabia. Before commencing the study, authorization was obtained from the author of the Arabic translator of the IdFAI [27]. This study obtained ethical approval from the Majmaah University Research Ethics Committee (number MUREC-Mar.27/COM-2024/12-2), which was approved on 27 March 2024.

### 2.2. Sample Size and Sampling Method

The study included 128 male participants aged 18 years and above who provided written informed consent and were currently playing volleyball without any clinical restrictions. Patients with a history of surgeries on musculoskeletal structures (i.e., bones, joint structures, and nerves) in either lower extremity were excluded. The sample size was set to 128 from a finite population size of 187 volleyball players in the Qassim region, using Taro Yamane’s formula: *n* = *N*/1 + *N* e^2^ [28]. Two-stage cluster sampling was used to recruit participants from eight volleyball clubs in and around the Qassim region. First stage: Five clusters (clubs) were chosen from the total eight clubs in the Qassim region using a simple random sampling method. Second stage: Within each cluster (club of interest), a subset of 128 participants was chosen using a simple random sampling method (16 participants from each club at random).

### 2.3. Study Procedure

This study was conducted in the Qassim region of Saudi Arabia, using a two-stage cluster sampling method. After an organized meeting with club managers, all individuals who met the inclusion criteria were invited to participate after completing a written informed consent form. Participant data was collected through face-to-face interviews with players in two phases. The first phase was a paper checklist that obtained demographic and anthropometric data to describe the study participants’ age, height, weight, Body Mass Index (BMI), as well as play and injury-related data such as training hours per week, playing duration in years, and dominant limb. Specific questions pertaining to associated variables of FAI, such as previous history of injury, reinjury, and episodes of “giving way”, as “yes” or “no” options, were included. The second phase involved distributing the Ar-IdFAI questionnaire to all the players. All players were informed of the questionnaire and the time it took to complete it. The questionnaire was distributed to 128 volleyball players who met the inclusion and exclusion criteria. For both ankle injuries (right and left), the participants completed the Ar-IdFAI scale independent of bilateral FAI. Data was collected for further analysis. Players were analyzed as individuals (totaling 128 datasets) rather than by the total number of ankles. No attrition, withdrawal rates, or missing data were observed.

### 2.4. Measurement Tool

The Identification of Functional Ankle Instability (IdFAI) questionnaire is a recently developed questionnaire that was specifically created to detect whether individuals meet a minimum requirement essential for inclusion in an FAI group [26,29]. Subsequently, the Identification of Functional Ankle Instability (Ar-IdFAI) questionnaire was validated in Arabic [28]. This questionnaire comprised 10 items. The first ankle was unrated, and the number of sprains on the same ankle (left or right) was counted. The other items were rated from 0 to 5, such as how often one feels ankle instability during sports or daily activities, or how often one’s ankle gives way [30]. Three distinct factors have been identified using IdFAI in previous studies. Factor 1 (history of ankle instability) included questions on the ability to stop the ankle from rolling over, the last time giving way episodes occurred, and the frequency of giving way episodes during sports or recreational activities. Together, these questions capture the entire feeling of ankle instability. Analysis of Factor 2 (the initial ankle sprain) involved questions about the number of sprains the player had, the duration since the last sprain, the period they required weight-bearing support, and the grade of the sprain diagnosed by a medical professional. It is believed that Factor 2 accurately depicted the degree of previous ankle sprain. This component was intended to offer a more accurate evaluation of the severity of previous ankle sprains. Questions about the amount of time that the ankle needs to heal after an ankle injury and questions about instability during activities of daily life comprised Factor 3. These inquiries revealed instability in day-to-day mobility functions. When instability occurs during routine activities, it may be severe instability that affects players frequently or every day, suggesting the existence of persistent instability [26]. The scores on the questionnaire ranged from 0 to 37, with a discrimination score of 10. As per the scale, an individual obtaining a total score of 11 or higher is classified as “instability,” while a score of 10 or lower indicates “no instability”. The greater the overall score, the more severe and prevalent the instability [27]. Injury-related variables (history of ankle injury, reinjury, and giving way) were examined as associated clinical indicators of FAI; however, their conceptual overlap with the IdFAI construct was considered during the interpretation of results.

### 2.5. Statistical Tools and Data Analysis

The data from the recruited volleyball players (*n* = 128) were analyzed using IBM SPSS Statistics version 27.0 (IBM Corp., Armonk, NY, USA). Normality of continuous variables was assessed using Kolmogorov–Smirnov and Shapiro–Wilk tests. Age, playing years, and weekly training hours showed significant deviation from normality (*p* < 0.001). Height and BMI showed minor deviations, but visual inspection of histograms and Q–Q plots indicated approximate normality. Weight was normally distributed (*p* > 0.05). Several continuous variables demonstrate deviations from normality; therefore, descriptive statistics are presented as median, interquartile range (IQR), and range. Categorical variables, including history of injury, reinjury, and episodes of “giving way,” are reported as frequencies and percentages.

Bivariate relationships between continuous variables and the presence of Functional Ankle Instability (FAI) were examined using the Mann–Whitney U test for non-normally distributed variables (age, height, playing years, and training hours per week) and Spearman’s test for normally distributed variables (Weight and BMI). Although several continuous variables deviated slightly from normality, independent-samples *t*-tests were applied to weight and BMI because these variables demonstrated approximately symmetric distributions within FAI groups and because the *t*-test is robust to moderate departures from normality, particularly with comparable group sizes (effect sizes were reported to aid interpretation). Associations between categorical injury-related variables and FAI were assessed using chi-square tests (or Fisher’s exact test where appropriate). Before multivariable modelling, multicollinearity was assessed using variance inflation factors (VIFs) and tolerance statistics obtained from a preliminary linear regression model. Height and weight demonstrated extreme multicollinearity with BMI (VIFs > 30–100) and were therefore excluded. Only BMI was retained as the primary anthropometric factor.

Multivariable binary logistic regression was then performed to identify independent associated factors with FAI. The dependent variable was dichotomized as FAI present or absent. Based on collinearity assessment and bivariate results, the final model included age, BMI, weekly training hours, and a selected injury-related variable (history of injury/reinjury/or episodes of “giving way”; only one was included per model to avoid collinearity). Model fit was evaluated using the Hosmer–Lemeshow goodness-of-fit test, and odds ratios (ORs) with 95% confidence intervals (CIs) were reported. Receiver Operating Characteristic (ROC) analysis using predicted probabilities was used to assess discriminatory performance (AUC).

Sensitivity analyses were performed by substituting alternative injury-related variables (e.g., reinjury instead of giving way) into the logistic model to evaluate the stability of associations. We anticipated the possibility of complete or quasi-complete separation in logistic regression models (regression analyses were therefore conducted for exploratory purposes, and coefficient stability was carefully evaluated).

## 3. Results

We recruited volleyball players from the Qassim region. A total of 128 volleyball players were included in the analysis. Visual inspection of histograms and Q–Q plots, supported by the Kolmogorov–Smirnov test, indicated deviations from normality for several continuous variables; therefore, medians, interquartile ranges (IQRs), and ranges were reported globally. Categorical variables were summarized using frequencies and percentages. Participants had a median age of 23 years and a median BMI of 23.8 kg/m^2^ and trained a median of 10 h per week (Table 1). Based on the Ar-IdFAI classification, 57 (44.5%) players met the criteria for Functional Ankle Instability (FAI). Of the 57 players, 43 players (75%) reported FAI in the right ankle, and 14 players (25%) reported FAI in the left ankle. Additionally, 100 players (78.1%) reported right-leg dominance, 89 (69.5%) had experienced at least one ankle injury, 54 (42.2%) had sustained an ankle reinjury, and 57 (44.5%) reported episodes of ankle “giving way” (Table 1). These figures highlight the persistent issues that volleyball players encounter and highlight how ankle instability may develop into chronic conditions during volleyball.

Upon bivariate association testing, non-parametric Mann–Whitney U tests revealed no significant differences between players with and without FAI in age (*p* = 0.514), BMI (*p* = 0.289), body weight (*p* = 0.115), playing years (*p* > 0.05), or weekly training hours (*p* = 0.699). The effect sizes were negligible. Spearman rank correlations between IdFAI scores and these variables were also minimal (ρ range: −0.09 to 0.06), indicating no meaningful linear or monotonic relationship between demographic or training-related factors and ankle instability (Table 2). Independent samples *t*-tests showed that athletes with FAI had significantly higher body weight than those without FAI (mean difference = 5.69 kg; *p* = 0.017; Cohen’s d = 0.43). This represents a moderate effect size. However, there was no significant difference in BMI between groups (*p* = 0.306; Cohen’s d = 0.18) (Table 2).

Because weight and height demonstrated extreme multicollinearity with BMI during diagnostic testing, weight and height were not included in subsequent multivariable regression models. Conversely, all injury-related variables demonstrated strong and statistically significant associations with FAI. Prior ankle injury, reinjury, and episodes of ankle giving way were significantly related to FAI (all *p* < 0.001). Effect sizes were large, with Cramér’s φ ranging from 0.59 to 0.64. These findings indicate that injury history and recurrent instability symptoms were substantially more common among athletes with FAI.

Strong associations were observed between injury-related variables and FAI. As expected, episodes of giving way demonstrated a perfect association with FAI (χ^2^ (1) = 128.0, *p* < 0.001, Cramér’s V = 1.00), reflecting the inclusion of giving way symptoms within the IdFAI classification framework. Previous injury (χ^2^ (1) = 45.03, *p* < 0.001, Cramér’s V = 0.59) and reinjury (χ^2^ (1) = 51.63, *p* < 0.001, Cramér’s V = 0.64) were also strongly associated with FAI. These effect sizes fall in the large-to-very-large range, indicating that historical injury burden, rather than demographic or training-related characteristics, is the primary factor associated with FAI. (Table 3).

Prior to logistic modeling, variance inflation factors (VIFs) were examined. Height and weight showed extreme multicollinearity with BMI (VIFs > 30–100). Consequently, height and weight were excluded from the regression analysis. Only one injury variable (previous injury) was included in this model to avoid further collinearity among injury-related factors.

The final multivariable logistic regression was conducted for exploratory purposes (the model included age, BMI, weekly training hours, and previous ankle injury as variables associated with FAI). The model demonstrated a statistically significant overall fit (Omnibus χ^2^ (4) = 66.17, *p* < 0.001) and good calibration according to the Hosmer–Lemeshow test (χ^2^ (8) = 5.739, *p* = 0.676). The model explained 54.0% of the variance in FAI (Nagelkerke R^2^ = 0.540) and correctly classified 79.7% of cases, indicating acceptable discriminatory performance; however, coefficient estimates for injury-related variables were unstable due to complete or quasi-complete separation. Consequently, these results should not be interpreted as evidence of independent variables associated with FAI (Table 4). Despite the computational limitations, their consistent statistical dominance of historical variables across models reinforces the conclusion that injury history and, particularly, recurrent symptoms such as giving way are the primary dominant associated features of FAI, while demographic and training-related characteristics do not independently contribute to risk.

In contrast to the injury variables, none of the demographic (age, BMI) or training-related variables associated with FAI (weekly training hours) significantly contributed to the model directly in the present sample. Specifically, age (*p* = 0.174), BMI (*p* = 0.903), and weekly training hours (*p* = 0.072) did not meaningfully predict FAI status once injury history was accounted for. The model’s discriminative ability was supported by the Receiver Operating Characteristic (ROC) analysis, which yielded an Area Under the Curve (AUC) of 0.855 (95% CI: 0.791–0.920). This result reflects excellent classification performance. Predicted probabilities derived from injury-related variables reliably differentiated players with and without Functional Ankle Instability (FAI), even though individual regression coefficients were destabilized by separation.

## 4. Discussion

To the best of our knowledge, this is the first study to report the prevalence of Functional Ankle Instability (FAI) among volleyball players using the IdFAI tool. In the present sample, 44.53% of players met the criteria for presence of FAI (right: 75%; left: 25%), and 69.53% reported at least one previous ankle sprain. Prior studies have shown that 75% of ankle sprains were initial occurrences, which was the most common type [17], which aligns closely and well with the high proportion of first-time sprains observed here. Ankle joints were the most affected and accounted for 36.6% of the injuries among volleyball players [31], and a recent study reported that 18% of Brazilian male volleyball players sustained severe injuries, with the ankle being the second most common [32,33]. In our study, 42.19% of players reported reinjury, consistent with the literature documenting reinjury rates between 12 and 47% [33]. Previous evidence consistently shows that athletes who experience a lateral ankle sprain are more susceptible to subsequent sprains [34], including those participating in volleyball [35]. Evidence has also shown that 75.38% of ankle sprains lead to multiple ankle sprains during sports activities [36].

The prevalence of “giving way” episodes in the present study was 44.53% (Table 1). This is markedly higher than the 19% prevalence reported among football players [12], which may reflect sport-specific demands. Volleyball requires high-energy and high-intensity activities such as running, cutting, direct contact, jumping, and landing, which result in frequent recurrence of ankle injuries [37,38]. Functional instability was evident, followed by poor proprioception, strength, and motor control [16,39]. A recent meta-analysis also found that ankle instability mainly occurs because of the sequelae of frequent ankle injuries, followed by altered proprioception [40]. The ankle joint is responsible for bearing excessive mechanical load because of the interaction between the ground and players, as well as direct contact with other players, and excessive pressure on the ankle or foot complex in athletes during the game also leads to various ankle injuries (micro or macro). Although previous studies in footballers have reported FAI prevalence of 41–46% using Cumberland ankle instability tool (CAIT) and IdFAI assessments [12], they did not examine the specific injury-related factors associated with FAI nor perform predictive modelling. In another study, factor 0.3% of football players reported FAI using the same IdFAI questionnaire; however, they did not establish an association between the injury-associated variables linked to FAI and did not analyze the regression model to confirm the variables associated with FAI. It has also been reported that initial ankle injuries lead to the recurrence of ankle injuries, which further results in FAI or episodes of “giving way” [41]. Evidence further shows that FAI leads to frequent reinjuries of the ankle joint and proprioceptive deficits [37,42]. To date, the prevalence of FAI among volleyball players has not been widely reported using the IdFAI tool, which is considered valid for discriminating between stable and unstable ankles. Therefore, FAI preventive measures are highly recommended among volleyballers. A previous study has also reported that ankle injuries and reinjuries can lead to ankle instability owing to a lack of proprioception and postural control, muscular weakness, and balance deficits with ligament laxities [20]. Ankle injuries, recurrence of ankle injuries [43], previous ankle injury [17,44], altered muscle patterns [38], episodes of ankle “giving way”, and multiple ankle sprains [37] are the most important variables associated with FAI. The interrelationship of these factors has been described as an injury sequence pathway, with fair-to-good associations among injury occurrences in footballers [12], and is supported by research demonstrating that recurrent ankle sprains often precipitate instability and subsequent giving way events [9].

The results showed no relationship between FAI and age, BMI, playing duration, training per week, or dominant limbs (R/L), except for height and weight. These findings align with evidence in football players showing similarly weak or nonsignificant associations for these variables [13,42]. Training-related variables such as practice duration or session frequency may vary considerably among athletes based on competition level, personal schedule, and positional demands, which may account for their inconsistent association with ankle instability [12,42]. The results of the present study also found no association between FAI and the dominant limb (R/L), which was also supported by the research conducted by Cruz and colleagues [9,12].

Although height, weight, and weekly training hours showed group differences in bivariate analyses, these variables did not show independent relevance once multicollinearity and model stability were accounted for. Height and weight demonstrated extreme multicollinearity with BMI, resulting in their exclusion from regression modelling, and none of the demographic or training-related variables showed significant direct associations with FAI in the present sample. Longitudinal and sport-specific biomechanical studies are needed to clarify the potential contextual and moderating roles of these variables, as these factors may influence ankle instability indirectly through mechanisms such as exposure load, movement patterns, fatigue, or neuromuscular control, which were not captured in the present analyses.

In contrast, all injury-related variables, including previous injury, reinjury, and episodes of giving way, exhibited strong and statistically significant associations with FAI (Cramér’s V = 0.59–1.00). Notably, giving way demonstrated perfect separation, with all athletes reporting giving way being classified as having FAI. These findings reinforce previous literature findings demonstrating that giving way is a hallmark symptom among individuals with chronic ankle instability [41,43]. Instability has also been attributed to proprioceptive deficits, altered neuromuscular control, and reduced postural stability among volleyball players on CAIT assessments [45], as factors such as neuromuscular control, lateralization, and functional symmetry further influence performance, particularly in ball-based team sport athletes (volleyball, handball, and basketball), who typically demonstrate faster reaction times across simple, optional, and cognitive tasks [46]. Previous studies also reported that 85.9% of sports players had a history of ankle sprain, of which 64.5% suffered from chronic ankle instability (CAI: An umbrella term that encompasses FAI as well), showing a strong association between the two [44]. Furthermore, ankle instability that develops from ankle sprains increases the risk of recurrence [47].

In our analyses, strong and significant associations among previous injury, reinjury, and giving way were observed (all *p* < 0.001), supporting the literature describing a cyclical pattern in which initial sprain increases susceptibility to further sprains and instability symptoms. However, because these variables produced near-complete or complete separation in logistic regression, they could not be reliably estimated as independent variables associated with FAI. Instead, their influence was expressed through strong bivariate associations rather than multivariable effect estimates. These patterns underscore that historical injury burden, rather than demographic or training-related characteristics, is closely linked to FAI.

The logistic regression model, which included only age, BMI, weekly training hours, and previous injury, demonstrated acceptable fit (Hosmer–Lemeshow *p* = 0.676) and moderate explanatory power (Nagelkerke R^2^ = 0.540). Although the multivariate logistic regression demonstrated strong discrimination, coefficient instability due to separation precludes interpretation of individual variables as independent variables associated with FAI. These findings should therefore be viewed as descriptive and exploratory rather than confirmatory. Previous research has also reported that ankle instability is strongly associated with greater height in young adults [47]. Another study reported that the history of multiple ankle sprains was associated with greater body mass, thereby reducing ankle stability [48]. However, none of the demographic or training-related variables associated with FAI reached statistical significance, and the coefficient for previous injury was unstable due to near-complete separation. The ROC analysis yielded an Area Under the Curve (AUC) of 0.855, demonstrating excellent internal discrimination between players with and without FAI within the study sample (absence of external or temporal validation here limits interpretation of the ROC analysis as a predictive tool), largely driven by the strong separation contributed by injury-related variables. Although this highlights the important clinical relevance of injury history, the instability of regression coefficients emphasizes caution when interpreting predictive contributions.

Collectively, this study established a link between injury burden and FAI prevalence in volleyball players, highlighting that recurrent injuries and giving way symptoms are strongly associated with ankle instability. These findings reinforce the importance of early rehabilitation following initial sprains and the need for preventive programs focusing on proprioception, neuromuscular control, and postural stability to reduce the progression toward functional instability and enhance volleyball performance.

### Limitations

The sample was limited to male volleyball players from sports clubs in the Qassim region of Saudi Arabia. Consequently, the findings cannot be generalized to players from other regions or female players, given that player characteristics may vary across clubs depending on their competitive level.

Given the cross-sectional design and the symptom-based nature of the IdFAI, all relationships described in this study should be interpreted as associations rather than causal or predictive effects.

Another important limitation of the present study is the conceptual overlap between certain injury-related variables (Specifically, previous ankle injury and episodes of giving way) and the IdFAI outcome measure, including the expected perfect association between giving way episodes and FAI classification. Consequently, the strong associations observed between these variables and FAI may be partially inflated due to shared construct domains rather than reflecting independent causal or predictive relationships. These findings should therefore be interpreted as descriptive associations that reinforce the clinical relevance of injury history within Functional Ankle Instability, rather than as evidence of independent associated variables or causal predictors of FAI.

These limitations highlight the need for longitudinal designs and external validation to further unravel temporal relationships between injury history and Functional Ankle Instability.

Despite these limitations, this is the first study to investigate the prevalence of FAI and the relationship between self-reported FAI and its associated variables among male volleyball players in the Qassim region. Further studies with larger and more diverse samples are required to validate and expand these findings. Researchers and coaches must be cautious when interpreting these results.

## 5. Conclusions

FAI was prevalent in 44.53% of male volleyball players. Injury-related characteristics, including previous ankle injury, reinjury, and perceived episodes of ankle “giving way,” showed strong associations with FAI, while demographic and training-related variables did not demonstrate direct associations within this sample. Given the cross-sectional design and the structure of the IdFAI, these findings should be interpreted as descriptive associations rather than evidence of independent predictors or causal relationships. Clinically, the results highlight the importance of early identification of instability symptoms and the implementation of preventive and rehabilitative strategies focusing on proprioception, neuromuscular control, and postural stability (indicators of FAI) to reduce progression toward Functional Ankle Instability in volleyball players.

## Figures and Tables

**Table 1:** Demographic dimensions/associated variables of the volleyball players recruited in the Qassim region (*n* = 128).

Demographic Dimensions	Median (IQR)	Range
Age (Years)	23 (19–32.8)	18 to 41
Height (cm)	176.5 (170–183.4)	160 to 198
Weight (kg)	73.5 (68–82)	43 to 118
BMI (kg/m^2^)	23.8 (21.5–25.7)	14.8 to 36
Playing duration (Years)	5 (3–10)	1 to 25
Training/week (hours/week)	10 (8–12)	4 to 24
FAI score	9 (0–17)	0 to 32
Dominant limb (R/L)	100 (78.1%)/28 (21.9%)	
Functional Ankle Instability (Y/N)	57 (44.5%, R = 43, 75%; L = 14, 25%)/71 (55.5%)	
Previous history of ankle injury (Y/N)	89 (69.5%)/39 (30.5%)	
History of reinjury (Y/N)	54 (42.2%)/74 (57.8%)	
Episodes of “giving way” (Y/N)	57 (44.53%)/71 (55.5%)	

Note: Demographic dimensions do not follow a normal distribution; hence, they are expressed as the median with an interval of interquartile range (IQR) and range. Additionally, right and left percentages indicate that the self-reported side predominantly affected among players is classified as having Functional Ankle Instability (FAI), not ankle-level prevalence.

**Table 2:** Comparison of demographic and training-related characteristics between FAI-positive and FAI-negative volleyball players using the Mann–Whitney U test. Independent samples *t*-test comparison of body weight and BMI between athletes with and without Functional Ankle Instability (FAI).

Variables	Group	Mean Rank	Mann–Whitney U	Z	*p*-Value
Age (years)	FAI Negative (*n* = 71)	66.63			
	FAI Positive (*n* = 57)	61.85	1872.5	−0.728	0.466
Height (cm)	FAI Negative (*n* = 71)	57			
	FAI Positive (*n* = 57)	73.84	1491	−2.560	0.01
Playing years	FAI Negative (*n* = 71)	66.46			
	FAI Positive (*n* = 57)	62.05	1884	−0.671	0.502
Training hours/week	FAI Negative (*n* = 71)	57.46			
	FAI Positive (*n* = 57)	73.27	1523.5	−2.439	0.015
Variables	Group	Mean ± SD	*p*-value	Effect Size (Cohen’s d)	t (df)
Weight (kg)	FAI Negative (*n* = 71)	72.46 ± 11.85			
	FAI Positive (*n* = 57)	78.16 ± 14.74	0.017	0.43 (small–medium)	2.42 (126)
BMI (kg/m^2^)	FAI Negative (*n* = 71)	23.55 ± 3.49			
	FAI Positive (*n* = 57)	24.20 ± 3.74	0.306	0.18 (negligible)	1.03 (126)

**Table 3:** Crosstabulation and chi-square analysis of the association between injury-related variables and FAI.

Injury Related Variables	FAI Negative (%)	FAI Positive (%)	χ^2^ (df = 1)	*p*-Value	Cramér’s V
Giving way episodes	No giving way: 71 (100%)	No giving way: 0 (0%)	128	<0.001	1
	Giving way: 0 (0%)	Giving way: 57 (100%)			
Previous ankle injury	No previous injury: 39 (100%)	No previous injury: 0 (0%)	45.03	<0.001	0.59
	Previous injury: 32 (36.0%)	Previous injury: 57 (64.0%)			
Ankle reinjury	No reinjury: 61 (82.4%)	No reinjury: 13 (17.6%)	51.63	<0.001	0.64
	Reinjury: 10 (18.5%)	Reinjury: 44 (81.5%)			

**Table 4:** Multivariable logistic regression model assessing demographic and training-related variables associated with Functional Ankle Instability (FAI).

Variables	B	SE	Wald	*p*-Value	OR (Exp(B))	95% CI for OR
Age (years)	−0.041	0.03	1.85	0.174	0.96	0.91–1.02
BMI (kg/m^2^)	0.008	0.067	0.015	0.903	1.01	0.88–1.15
Training hours/week	0.125	0.069	3.245	0.072	1.13	0.99–1.30
Previous ankle injury	−21.986	6275.417	0	0.997	0	0.000–∞ *
Constant	0.116	1.735	0.004	0.947	1.12	—
Age (years)	−0.041	0.03	1.85	0.174	0.96	0.91–1.02

Note: The extremely large SE and OR approaching 0 reflect quasi-complete separation, where previous injury nearly perfectly distinguishes FAI-positive from FAI-negative players. The coefficient cannot be reliably estimated. Accordingly, the model does not support inference regarding independent variables associated with FAI, * Correlation is significant at the 0.05 level (2 tailed).

## Data Availability

The dataset that was analyzed or the results presented in this study are available upon reasonable request from the corresponding author.

## Abbreviations

The following abbreviations are used in this manuscript:

FAI	Functional Ankle Instability
IQR	Interquartile Range
BMI	Body Mass Index
FI	Functional Instability
ROM	Range of Motion
PROMs	Patient-Reported Outcome Measures
IRB	Institutional Review Board
IBM	International Business Machines
SPSS	Statistical Package for the Social Sciences
Ar-IdFAI	Identification of Functional Ankle Instability
KS	Kolmogorov–Smirnov test
cm	Centimeter
kg	Kilogram
CAIT	Chronic Ankle Instability Tool
CAI	Chronic Ankle Instability
